# Supplementary material for: Cutaneous information processing differs with load type during isometric finger abduction
Source: PLoS One. 2022 Dec 22;17(12):e0279477. doi: 10.1371/journal.pone.0279477 (PMC9778995; doi:10.1371/journal.pone.0279477)
Supplement: Attachment — Submitted filename: Response to Reviewers.docx [file pone.0279477.s002.docx]

Dear Editor and Reviewer

Thank you very much for reviewing our manuscript and offering valuable advice.

We have addressed all the comments with point-by-point responses and revised the manuscript accordingly.

1. The authors argued that cutaneous information processing differs based on differences in somatosensory evoked potentials in response to cutaneous nerve stimulation. However, the manuscript does not discuss this result in depth, and the physiological implications derived from this result are minimal. In particular, only N33 was attenuated in this study, but the authors did not explain what physiological significance lies behind this. It is also necessary to consider why N33 is attenuated while other components remain unchanged. Simply stating that cutaneous information processing differs depending on the isometric tasks is not novel compared to previous studies.
**Response:** Thank you for raising an important point. We have added an explanation of the physiological implications of the finding that N33 amplitude was attenuated to the Discussion (Page 12-13, Line 305-312).

2. The authors strongly argued that the greater the attenuation of the SEP, the more sensory input is needed under those conditions. However, in my view, a decrease in S1 response means that less sensory information reaches the S1, i.e., that the S1 does not need somatosensory signals from the periphery. Therefore, the authors should provide more evidence or explanation why more sensory information is needed under conditions of greater SEP attenuation.
**Response:** We appreciate your suggestion. A decrease in SEP amplitude during the motor task compared to rest, that is, SEP gating, does not imply decreased S1 excitability. The functional significance of SEP gating is thought to be that it suppresses nerve excitability caused by electrical stimulation, which is necessary for recording SEPs but constitutes a source of noise during task execution. Please refer to Page 4, Lines 86 to 89 for this content.

3. The result that cutaneous-muscular reflexes are enhanced has already been described in a paper (Gibbs et al 1995). Therefore, this study lacks novelty; it is necessary to claim the difference from the results of Gibbs et al.
**Response:** We agree that the result that the E2 amplitude of CMR was larger in muscles performing voluntary contraction than in posturally active muscles has been reported by Gibbs et al. However, we suggest that our study has novelty in the following two points.

1. Gibbs et al compared the amplitude of CMR recorded from lower and trunk muscles following electrical stimulation of the digital nerves of the second toe between the voluntary task (subjects lay supine or prone and voluntarily contracted lower limb and trunk muscles) and postural task (subjects maintained an upright posture). However, in the present study, we compared the amplitude of CMR between two tasks that differed only in pure load type.

2. We simultaneously measured SEPs and found that cortical responses to cutaneous information differed between the two tasks.

4. The authors only explain the isometric tasks, and there is little background information to consider why differences in SEPs occur. Since the authors measured muscle activity, they should show muscle activity between the two tasks. Since in the position task co-contraction of agonistic muscle pairs occurs, it is inferred that whole muscle activity is larger in the position task than the force task. This would be consistent with a previous study that showed that greater muscle activity is associated with greater sensory gating (Sugawara et al., 2016). We would like to know if the differences in SEP modulation simply reflect differences in the magnitude of muscle activity.

**Response:** Thank you for pointing out an important aspect. We have noted that subjects performed the position and force tasks for approximately 90 s (divided into three blocks of 30 s) each before recording the SEPs and CMR, and confirmed that EMG activity of the right FDI muscle was equal between both tasks in the sub-section of “Protocol” (Page 7, Line 172-174). Furthermore, we monitored the FDI EMG activity during the recordings of SEPs and CMR to ensure that the EMG activity was equal between the two tasks. This explanation has been added to the manuscript (Page 8, Line 186-187).

5. Introduction is not easy to understand.
1. In the first paragraph, the authors described that it is difficult to evaluate Ia and Aβ separately (lines 63-65). However, in the third paragraph, they described that many experiments have been done to stimulate cutaneous nerves (lines 79-82). These sentences are not consistent. The text should be rearranged.

**Response:** Thank you for pointing out that our statement could be understood in this way. In daily life, both Aβ and Ia fibers are activated by movements and these two kinds of information are important for precise movement execution. Therefore, it is difficult to set up an assignment that isolates their respective contributions. In the present study, we addressed this issue by using isometric contraction tasks with different load types under experimental environments. We have rearranged these sentences (Page 3, Line 63-65).

2. In the first sentence of the second paragraph, the authors described that modality specific information processing is being investigated. But, in fact, only muscular information processing is mainly mentioned in the paragraph.

**Response:** Thank you for pointing this out. We evaluate not only muscle information but also reflex responses of the spinal cord or central nervous system to nerve stimuli (especially group Ia fibers) by EMG. We highlighted that only proprioceptive information processing has been found to differ between the position and force tasks (Page 3, Line 66-67).

3. Before the last sentence of the second paragraph, it is necessary to state the reason why cutaneous information processing is examined in the position task and the force task.

**Response:** As pointed out, we have described the importance of examining differences in cutaneous information processing between the position and force tasks (Page 4, Line 78-82).

Minor points
Lines 216-218; The authors should be more specific about the measurement of SEP.
**Response:** Thank you for your helpful advice. We have provided a more detailed description of the analysis of SEP data (Page 9, Line 224-225 and 227-228). Further descriptions of SEP measurement can be found in the sub-section “Recordings of SEPs”.

Lines 295-301; Doesn't the greater attenuation of SEP to peripheral stimuli mean that the central nervous system does not need that information?
**Response:** This is correct. We have inserted material about the functional role of SEP gating into the Discussion (Page 13, Line 312-321)

Lines 337-347; Since the visual feedback is the same in the two tasks, there is no need to discuss the influence of visual feedback on somatosensory processing. This paragraph should be deleted.
**Response:** As pointed out, we have deleted that paragraph.

Does the noise affect the results because the SEP signal is noisy in Figure 3. Does applying a 200 Hz low pass filter not change the results?

**Response:** We appreciate your suggestion. While we agree that restricting the low-pass filter would reduce the noise, we prefer to keep the low-pass filter in its present configuration because restriction would also affect the amplitude of the short latency components of SEP (Desmedt et al., 1974; Shaw, 1992). As you pointed out, the SEP raw waveform in Fig. 3 may look a little noisy, but this presents no problems for measuring the important amplitude values.
