# Supplementary material for: Cutaneous information processing differs with load type during isometric finger abduction
Source: PLoS One. 2022 Dec 22;17(12):e0279477. doi: 10.1371/journal.pone.0279477 (PMC9778995; doi:10.1371/journal.pone.0279477)
Supplement: Attachment — Submitted filename: Response to Reviewers.docx [file pone.0279477.s003.docx]

Dear Editor and Reviewer

Thank you very much for reviewing our manuscript and offering valuable advice.

We have addressed all the comments with point-by-point responses and revised the manuscript accordingly.

1. Although the function of sensory gating is said to reduce irrelevant information, the authors’ experiment did not show whether the sensory information was needed in such a situation. Without careful explanation, it is shortsighted to argue that a situation indicating the greater SEP gating requires more sensory information. The related sentences should be removed (lines 92-95, 286-287).

**Response**: As pointed out, we have not demonstrated that the afferent information from the finger nerves is important or that the electrical stimulation to elicit SEP is noise. Thus, we have deleted the sentences you pointed out and revised several sentences in the Discussion (Page 13, Line 308-316; Page 15, Line 366-367).

2. The authors should add a discussion which argues a difference between a previous paper about the enhancement of cutaneous-muscular reflexes (Gibbs et al 1995), as the authors responded as the first point.

**Response**: As suggested, we have described in detail the results of Gibbs et al. in order to clarify the difference between the present and previous studies (Page 14, Line 340-344).

3. The author stated that the position and force tasks require a similar net muscle torque (Lines 70-71). However, they only recorded the activity of a single muscle (FDI). We do not know whether the total activity of the whole hand muscles is equivalent between two tasks. A previous study showed that greater muscle activity is associated with greater sensory gating (Sugawara et al., 2016). The difference in sensory gating between the two tasks may simply reflect differences in the magnitude of muscle activity. As it stands, this paper may send the wrong message. Therefore, the authors should add a discussion of the possibility that differences in muscle activity that are not recorded cause differences in sensory gating between two tasks.

**Response**: Thank you for pointing out an important aspect. We have discussed about this point in the Discussion (Page 14-15, Line 355-360).
